# Latent profiles for mental health in older people from Concepción, Chile.

**DOI:** 10.1192/j.eurpsy.2023.542

**Published:** 2023-07-19

**Authors:** S. Saldivia, J. Aslan-Parra, C. Bustos, C. Inostroza, F. Cova, A. Castillo

**Affiliations:** ^1^ Psychiatry and Mental Health; ^2^Universidad de Concepcion, Concepción, Chile; ^3^Psychology; ^4^Fundamentos de Enfermería y Salud Pública, Universidad de Concepcion, Concepción, Chile

## Abstract

**Introduction:**

Aging is a demographic global trend and a challenge for public mental health; however, gaps persist for a comprehensive definition of mental health, risk, protective factors, and processes involved, which represent a greater problem in middle-income countries, where evidence is scarce.

**Objectives:**

To identify combined mental health profiles in older adults, based on self-report of anxiety symptoms, depressive symptoms, and perception of well-being, and to identify risk and protective variables for each of the groups, based on a sample of older adults attending primary health care (PHC) centers in the Province of Concepción, Chile.

**Methods:**

A convenience sample of 573 adults of both sexes, over 65 years, autonomous, attending PHC centers in the Province of Concepción, Chile, answered a set of instruments assessing anxiety symptoms (SCL-90), depressive symptoms (PHQ-9) and perception of well-being (Pemberton Happiness Index) and eventually associated variables that included sociodemographic and living arrangements, social participation, threatening life events (LTE), loneliness (ULS-3), and social support (MSPSS). Latent profile mixture analysis was used to identify groups of adults with similar mental health, and pertinence in each group was explained using random forests. The relationship between predictors and latent profiles were analyzed with multinomial regression.

**Results:**

A solution of 4 groups with distinctive mental health profiles was determined: Group 1 (28%) with high depressive symptoms, high anxiety, and low well-being; Group 2 (32%) with moderate depressive symptoms, high anxiety and moderate well-being; Group 3 (24%) with moderate depressive symptoms, low anxiety and moderate well-being and; Group 4 (15%) characterized by individuals with low anxious or depressive symptoms, high well-being, and absence of mental disorder.

Using random forests, this model predicts 63% variance between groups. A large number of variables were found to significantly predict membership in one of the 4 groups. Specifically: gender, satisfaction with living arrangement, economic crisis, own disease, and death or illness of friend, perception of general health, intimate, relational and collective loneliness, social support from family and significant others, and social support from friends.

**Conclusions:**

The 4-group classification is a parsimonious solution where group 1 characterize people with poor mental health; groups 2 and 3 languishing with high and low anxiety respectively; and group 4 healthy and flourishing. Overall, these groups highlight the role of close interpersonal relationships or primary ties, both in terms of intimacy versus loneliness/isolation and in satisfaction with living arrangements for the elderly. The importance of these psychosocial predictors on combined mental health in the elderly further the need to understand their role and mechanisms to design promotion and prevention strategies.

**Disclosure of Interest:**

None Declared

